# A research roadmap for AI opportunities in student assessment for medical education

**DOI:** 10.1186/s12909-025-08078-7

**Published:** 2025-11-26

**Authors:** Morteza Rezaei-Zadeh, Magdalena Cerbin-Koczorowska

**Affiliations:** 1 University of Leicester, Leicester, UK; 2 University of Edinburgh Medical School, Edinburgh, United Kingdom

**Keywords:** Artificial intelligence, Medical education, Student assessment, AI opportunities

## Abstract

The integration of Artificial Intelligence (AI) in medical education is rapidly transforming assessment practices, offering unprecedented opportunities to enhance student evaluation, feedback, and learning pathways. However, despite the potential, a comprehensive understanding of these opportunities and their interdependencies has been lacking. This study provides a critical review of the literature on AI’s role in medical education assessment, categorising 22 identified opportunities into seven major “mega-opportunities” that address various aspects of student assessment. Through the application of Interpretive Structural Modelling (ISM), the cause-effect interdependencies among these mega-opportunities were explored, revealing a complex web of relationships that guide their effective implementation. The findings highlight the central role of “Automated Feedback and Evaluation” and “Data-Driven Analytics and Curriculum Improvement” as foundational drivers, with far-reaching impacts on other areas like “Simulation-Based Assessment” and “Longitudinal Assessment and Development.” This paper culminates in the proposal of a*research roadmap* that highlights the priority of addressing different mega-opportunities in AI and assessment, offering practical guidelines for medical researchers, educators, institutions, and policymakers to adopt AI-driven assessment strategies. Future research avenues are identified to explore the real-world application and impact of these AI-driven innovations, focusing on longitudinal studies and educational equity. The findings underscore the need for continued research to refine the model proposed by this study and adapt it to diverse educational environments.

## Introduction

 Artificial intelligence (AI) is reshaping the landscape of student assessment, offering novel opportunities for enhancing accuracy, efficiency, and personalisation across various stages of the assessment process - impacting students, markers, moderators, and institutional bodies such as boards of studies. In higher education, AI is increasingly integrated into a range of assessment practices. This includes summative assessments - such as automated scoring and adaptive testing [1]; formative approaches, including real-time feedback systems; and programmatic strategies, where machine learning–powered predictive analytics can identify students at risk of underperformance with up to 88% accuracy, enabling early intervention and personalised support [2]. In this article, we understand AI broadly as algorithmic computer systems capable of performing tasks that typically require human intelligence [3], applied across these three assessment domains. As the global AI in education market is projected to surpass from USD 4.8 billion in 2024 to USD 75 billion by 2033 [4], these innovations signal a profound shift in how educators approach data collection and utilisation to support personalised growth and make reliable judgements about student’s performance —raising both exciting possibilities and complex ethical considerations.

These issues received considerable critical attention within the field of medical education (ME), where decisions concerning students’ competence and readiness for practice carry profound implications for addressing healthcare needs and patient safety. Despite significant progress observed in ME assessment practices over recent decades, training programs continue to encounter challenges that hinder effective assessment practices [5].

Traditional assessment methods - such as multiple-choice quizzes, written essays, and even clinical evaluations - often fall short in capturing the breadth and depth of competencies required for medical practice [6]. These methods tend to focus heavily on knowledge recall, frequently neglecting higher-order cognitive skills such as clinical reasoning, communication, and ethical decision-making [7]. Furthermore, issues like subjectivity in scoring, variability among assessors, and lack of timely, actionable feedback persist across many medical education contexts [8]. Given these longstanding challenges, various stakeholders emphasise the urgent need for more adaptive, scalable, and personalised approaches to assessment using AI [9]. While AI presents transformative opportunities, some caution against overreliance on EdTech in sensitive areas like education and assessment [10].

Recent advances in AI offer promising solutions to many of the above-mentioned challenges. AI-powered tools, including Natural Language Processing (NLP), Machine Learning (ML) algorithms, and Intelligent Tutoring Systems (ITS), are increasingly being used to provide real-time, individualised feedback, simulate clinical decision-making environments, and evaluate complex learner behaviours [11, 12]. Additionally, AI supports scalability by automating grading and offering adaptive testing models that respond to the learner’s performance in real-time [13]. These capabilities are seen as a potential opportunity to improve the precision and fairness of assessments while aligning with the broader shift toward data-driven precision education [14]. Despite the growing body of research exploring AI in medical education, current literature on AI’s role in student assessment remains fragmented and descriptive, often focusing on isolated use cases or theoretical potential without synthesising broader trends or outcomes [15, 16]. While several studies highlight specific opportunities - such as automated scoring, adaptive testing, or diagnostic simulations - few offer a comprehensive framework that connects these developments or evaluates their interdependencies. Moreover, existing reviews often overlook the strategic implications of AI for long-term assessment reform, especially in relation to competency-based education and ethical evaluation practices [17]. This gap calls for a systematic, critical review that not only maps existing opportunities but also models their causal relationships and provides a future-oriented roadmap for research and practice.

Building upon the identified gap, this paper aims to provide a comprehensive and critical review of the literature on the opportunities that AI offers for student assessment in medical education. By systematically categorising existing studies and modelling the interdependencies among the reported opportunities, the study seeks to move beyond descriptive analysis and offer an integrative understanding of how AI is reshaping assessment practices in medical education. In doing so, it also offers a forward-looking roadmap to guide future research and practical implementation. To achieve these aims, this study addresses the following research questions (RQs):

RQ1: What opportunities does AI offer for student assessment in medical education?

RQ2: What are the interdependencies and structural relationships among these identified opportunities?

RQ3: How can these relationships be leveraged to develop a strategic roadmap for implementing AI in medical education assessment?

The paper is structured as follows: First, it outlines the methodological approach used in the literature review and modelling. Next, it presents the key categories of AI-driven assessment opportunities identified across the literature. This is followed by a discussion of the interrelationships among these categories and their broader implications. The paper concludes with a critical reflection on existing gaps and offers recommendations for future research directions and policy considerations.

## Methodology

This study adopts a qualitative, exploratory research design [18], employing a Systematic Literature Review (SLR) complemented by thematic synthesis and causal modelling using Interpretive Structural Modelling (ISM) to explore and conceptualise the opportunities that AI brings to student assessment in medical education.

### Research design

Given the dynamic and rapidly evolving nature of AI technologies in education, particularly in student assessment, an SLR was selected as the primary method to synthesise existing knowledge. To further explore the interrelationships among the identified opportunities, the synthesis was followed by the application of ISM. ISM is a well-established method used to analyse complex systems by identifying and structuring interdependencies among elements. Its relevance in educational and healthcare research lies in its ability to construct hierarchical models that help visualise how certain opportunities may act as enablers for others, providing strategic insights for implementation [19, 20]. In this study, ISM was used to develop a four-layered model that illustrates the interconnectedness and influence pathways among AI-driven opportunities in medical student assessment.

#### Search strategy and inclusion criteria

**Fig. 1:**
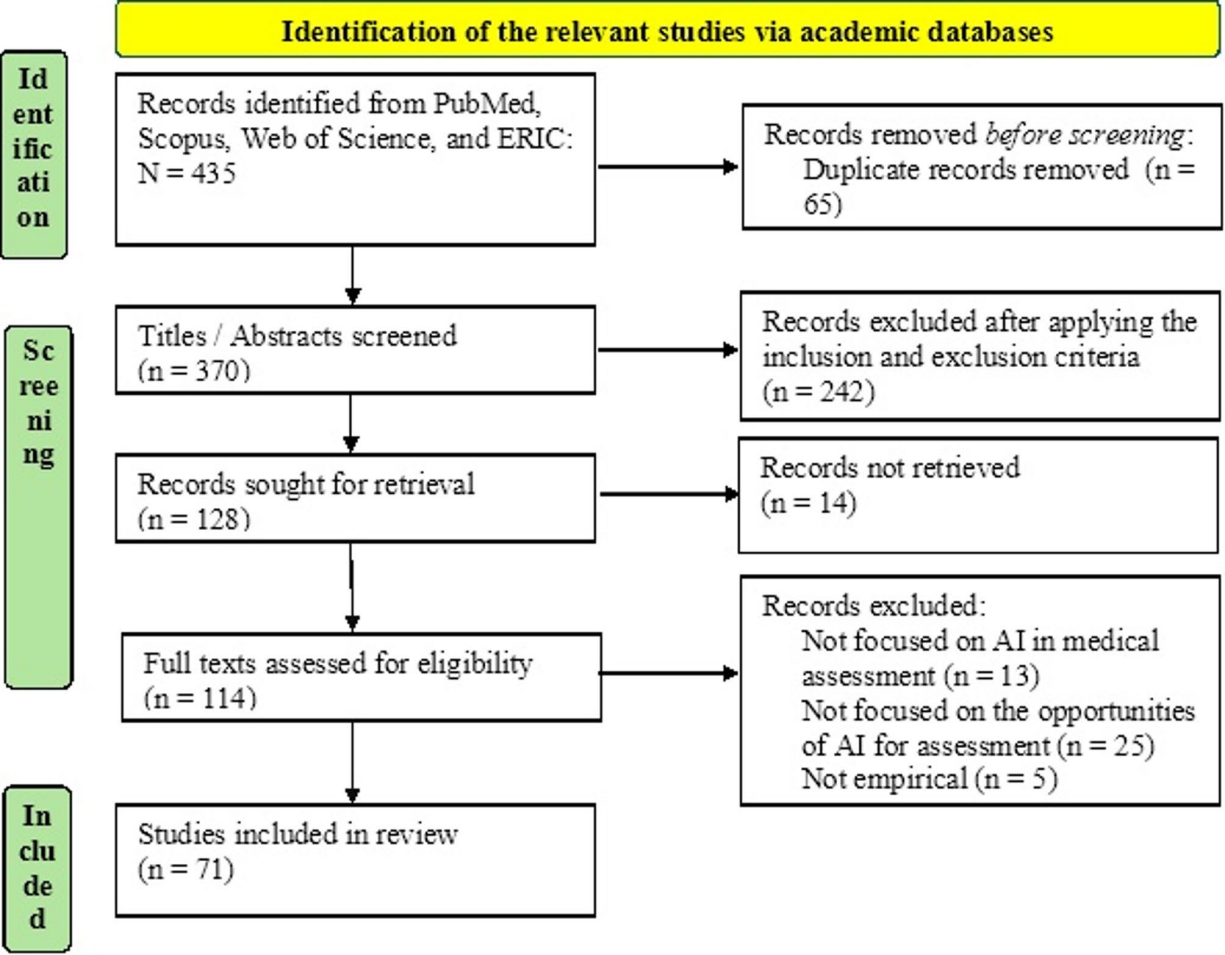
PRISMA diagram of the systematic review conducted by this study

Figure 1 summarises the flow diagram for the systematic review conducted by this study.

As outlined in Fig. 1, a systematic search was conducted across PubMed, Scopus, Web of Science, and ERIC databases using a PCC (Population, Concept, Context) question as follows: What opportunities does AI offer for student assessment in medical education? The search strategy involved combining search terms using Boolean operators. Terms within each category were linked with ‘OR’, and the three categories were linked with ‘AND’. Search terms were applied to the title and abstract fields of each database. The search covered studies published between 2010 and 2025 to capture the recent advancements in AI and its applications in medical education assessment.

A comprehensive list of search keywords is presented in Table 1.

**Table 1:** Search terms used in the systematic review

Category	Search Terms
Population	“medical education” OR “health professions education” OR “medical students”
Concept	“artificial intelligence” OR “AI” OR “learning analytics” OR “machine learning”
Context	“student assessment” OR “evaluation” OR “summative assessment” OR “formative assessment” OR “adaptive testing”

#### Screening and data extraction

A total of 435 studies were initially identified. After removing 65 duplicates, 370 records remained. Following a title and abstract screening based on the inclusion and exclusion criteria, 252 studies were excluded, leaving 118 articles for full-text review. Of the 118 articles selected for full-text assessment, 14 were excluded due to unavailability of the full text. The remaining 104 full-text articles were reviewed in detail against the inclusion and exclusion criteria. As a result, 43 studies were excluded for not meeting the criteria, leaving 71 studies that were included in the final systematic review.

Inclusion criteria were: (1) Peer-reviewed journal articles, (2) Studies focused on AI applications in student assessment, (3) Context: undergraduate, postgraduate, or continuing medical education, (4) Published in English, (5) Empirical, conceptual, or review studies. (6) Published after 2010.

Exclusion criteria included papers that: (1) Focused solely on AI applications outside assessment (e.g., diagnosis training), (2) Did not address medical education or health professions, (3) Were editorials or opinion pieces without substantial data.

### Thematic synthesis and categorisation

A reflexive approach to thematic analysis, a method known for its flexibility and rigour in identifying patterns within qualitative data [21], was employed. The researchers’ active role throughout the analysis process is emphasised by this approach, and our interpretations and perspectives were acknowledged as central to theme development. The process began with a thorough familiarisation with the literature, after which the identified opportunities for AI in medical student assessment were iteratively coded. Coding was conducted collaboratively by the two researchers, and themes and coding decisions were regularly discussed and refined to ensure consistency and consensus. Through this initial coding process, a total of 22 distinct opportunities were identified. These initial codes were then reviewed and refined, and through a process of constant comparison and synthesis, they were grouped into seven overarching themes, which were termed ‘mega-opportunities’. Each mega-opportunity reflects a broader, more abstract theme of AI’s potential to address specific challenges within medical education assessment, grounding the findings in the practical realities of the field.

### Causal modelling with interpretive structural modelling (ISM)

To explore the significant interdependencies among the seven identified mega-opportunities of AI in medical education assessment, this study employed ISM. ISM is a structured collective intelligence methodology that helps to identify and model complex relationships among components of a system by building a multilevel hierarchical structure [22]. This approach was specifically chosen for its ability to transform expert qualitative judgment into a hierarchical model, which was ideal for this study’s exploratory and conceptual nature. Unlike quantitative methods like Structural Equation Modelling, ISM is uniquely suited for mapping complex interdependencies based on collective intelligence rather than large datasets. Rather than analysing all possible influences among the mega-opportunities, ISM was specifically used to uncover the significant and structurally influential relationships, as determined through expert judgement and consensus during the structural self-interaction matrix (SSIM) development phase. This approach, which aligns with established ISM methodology [22], helps mitigate bias by relying on a transparent and collaborative decision-making process involving domain experts, thereby offering a practical and theoretically grounded roadmap for implementation and future research.

A panel of seven experts from three academic institutions was purposefully selected to participate in the ISM process. The inclusion criteria were: (1) a minimum of five years of professional experience in medical education, (2) direct involvement in the assessment of medical students for at least three academic years, and (3) demonstrated experience with integrating AI in medical education or research, evidenced by at least one peer-reviewed publication, conference presentation, or implementation project. These experts were invited to engage in a structured ISM session during which they responded to pairwise comparison questions generated by the ISM software. The expert judgment was collected through an online, synchronous session where a facilitator guided the panel through the pairwise comparisons. Consensus was achieved in real-time through facilitated discussion, where experts were encouraged to share their rationale for their judgments, especially in cases of initial disagreement. Final decisions for each relationship were then determined by a majority of expert judgments. The core question used to populate the Structural Self-Interaction Matrix (SSIM) was: “Does Mega-opportunity X significantly impact Mega-opportunity Y?”

Following the completion of the SSIM, the ISM process proceeded through standard stages: (1) development of the initial and final reachability matrices, (2) partitioning of levels, and (3) generation of a directed graph (digraph) representing the hierarchical relationships among the mega-opportunities [23]. The final output was a four-layer hierarchical model, clarifying how some mega-opportunities function as foundational drivers while others are outcomes or dependencies within the system. This modelling approach supports better strategic planning and theoretical understanding of AI’s role in reshaping medical education assessment.

### Critical appraisal and validation

To ensure the reliability and validity of the results, the quality of the studies included in the review was appraised using the Mixed Methods Appraisal Tool (MMAT) [24]. In addition, the thematic synthesis and ISM modelling were reviewed by three experts in the fields of AI, educational assessment, and medical education, providing feedback to enhance the robustness of the findings.

## Findings

The findings of this study are divided into two main sections: (1) Identification and categorisation of AI opportunities in medical education assessment, (2) Modelling the cause-effect interdependencies among those opportunities.

### Identification and categorisation of AI opportunities in medical education assessment

The first stage of this study involved conducting a comprehensive literature review to identify and synthesise the diverse opportunities that AI presents for student assessment in medical education. Through an iterative and thematic analysis of the 71 studies included in this review, 22 distinct opportunities were extracted from recent studies, reports, and academic discussions in the field. These opportunities encompass various functions, ranging from personalised learning and competency tracking to simulation-based assessment and curriculum enhancement.

To bring conceptual clarity and manageability to this diverse set of opportunities, a thematic categorisation process was applied. As a result, the 22 opportunities were grouped into seven inclusive categories, hereafter referred to as mega-opportunities. Each mega-opportunity represents a broader functional domain in which AI technologies are transforming the practice of assessment in medical education. These mega-opportunities are as follows: (1) Personalised Learning and Adaptive Assessment, (2) Automated Feedback and Evaluation, (3) Simulation-Based Assessment, (4) Competency-Based and Skills Assessment, (5) Data-Driven Analytics and Curriculum Improvement, (6) Enhancing Focus, Efficiency, and Time Management, (7) Longitudinal Assessment and Development. 

Below, each mega-opportunity is defined and the specific opportunities within it are listed in Table 2.

**Table 2:** Mega-opportunities of AI for medical student assessment

Mega-Opportunity	Definition	Corresponding Opportunities	References
Personalised Learning and Adaptive Assessment	AI technologies that tailor assessments, learning paths, and feedback to the individual needs, strengths, and weaknesses of students, enabling a more personalised educational experience.	Personalised Learning Pathways and Remediation	[25–27]
		Predictive Analytics for Student Performance	[28–31]
		Automated Generation of Assessment Items	[32–35]
		Medical Students’ AI-driven Self-assessment	[36, 37]
		AI for Automated Generation of Personalised Learning Artifacts	[38–41]
Automated Feedback and Evaluation	AI-driven software that provide timely and automated feedback on student performance, helping students identify their strengths and areas for improvement with precision and efficiency.	Automated Feedback on Medical Procedural Skills	[42–45]
		Automated Scoring of Open-Ended Questions and Essays	[46–49]
		AI-Driven Personalised Feedback on Documentation Skills	[50–53]
Simulation-Based Assessment	AI technologies that enhance the simulation experience by offering realistic clinical scenarios and detailed feedback on student performance, including both cognitive and procedural skills.	Simulation-Based Assessment with AI Feedback	[54–59]
		AI-Enhanced Simulation Fidelity and Realism	[56, 58, 59]
		AI-Assisted Standardised Patient Encounters	[60–64]
Competency-Based and Skills Assessment	AI systems designed to track and assess whether students meet specific clinical, academic, non-technical, and professional competencies, ensuring they are prepared for real-world practice.	Analysis of Communication Skills	[65, 66]
		AI-Driven Competency-Based Assessment	[67–69]
		AI-Driven Analysis of Medical Imaging for Competency Assessment	[70–73]
		AI-Assisted Standardised Patient Encounters	[60–64]
		AI-Enhanced Clinical Reasoning Assessment	[52, 62, 74]
Data-Driven Analytics and Curriculum Improvement	AI-driven tools that collect and analyse data from assessments, performances, and other learning activities to help identify trends, monitor student progress, and improve educational curricula.	AI-Driven Analysis of Learning Analytics Data for Curriculum Improvement	[75–77]
		Analysis of Clinical Performance Data	[78–81]
		AI-Driven Analysis of Multimodal Data for Holistic Assessment	[82–85]
Enhancing Focus, Efficiency, and Time Management	AI tools that support students in managing their study schedules, optimising focus, and improving time management, ultimately aiding in more effective goal setting and task execution.	Adaptive Testing	[86–89]
		AI-Enhanced Clinical Reasoning Assessment	[52, 62, 74]
		AI-Facilitated Feedback on Team-Based Learning	[90, 91]
		AI-Assisted Remote Clinical Skills Assessment	[92–94]
Longitudinal Assessment and Development	AI systems that support long-term tracking of students’ academic, clinical, and professional development over time, helping to monitor progress toward mastering competencies and achieving career goals.	Personalised Learning Pathways and Remediation	[25–27]
		AI-Facilitated Continuous Professional Development (CPD) Assessment	[95–98]
		AI-Assisted Standardised Patient Encounters	[60–64]
		Automated Generation of Assessment Items	[32–35]

The table above provides a structured synthesis of the diverse ways in which AI is being utilised to enhance student assessment in medical education. Each of the seven mega-opportunities represents a broader functional area where AI demonstrates transformative potential ranging from personalised learning to longitudinal development. By clustering the 22 individual opportunities into these thematic categories (mega-opportunities), the table not only offers conceptual clarity but also highlights overlapping areas of impact (e.g., some opportunities like AI-Assisted Standardised Patient Encounters appear under multiple mega-opportunities). This categorisation serves as the analytical foundation for the next phase of the study, where these mega-opportunities are further examined for their interrelationships and causal dynamics using ISM. The clarity provided by this framework is particularly critical in a field where AI’s applications are rapidly evolving and often fragmented across various studies and contexts.

### Modelling the interrelationships among mega-opportunities using ISM

**Fig. 2:**
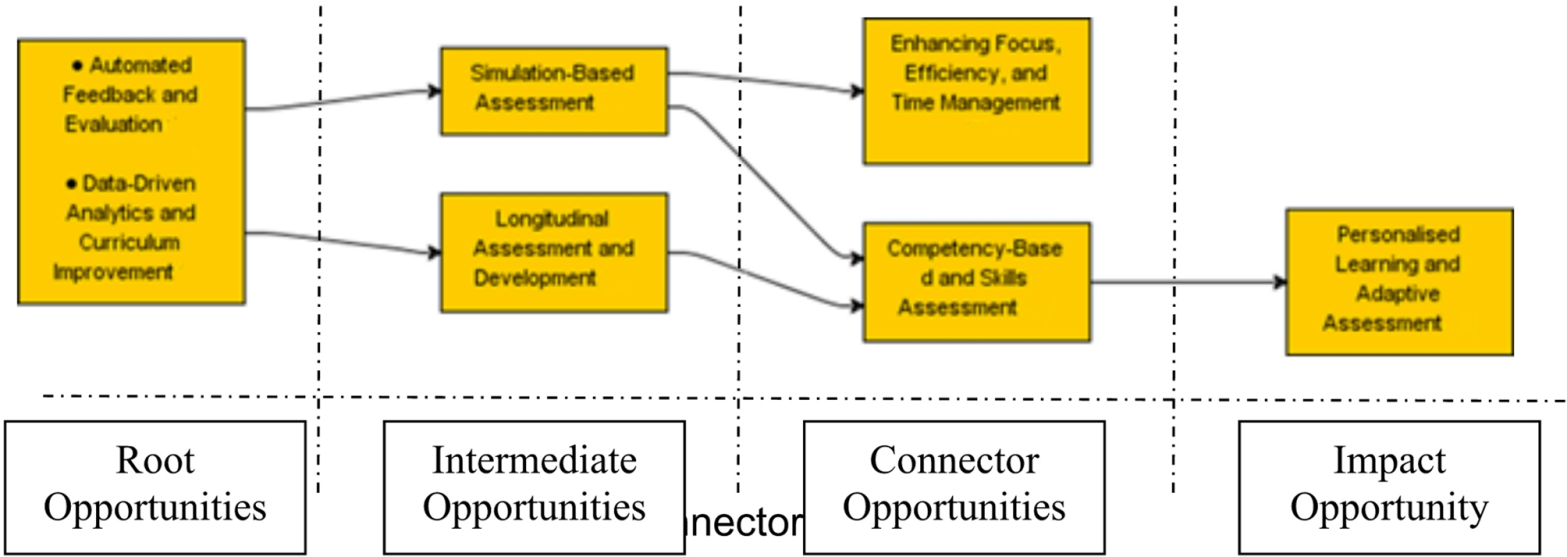
The interdependency model among the seven mega-opportunities of AI for medical education assessment

Building on the categorisation of 22 AI-related opportunities into seven mega-opportunities, this section presents the outcome of the ISM-based modelling that explored their cause-effect interdependencies. The aim was to uncover how these mega-opportunities influence one another and to construct a hierarchical framework that reflects their strategic significance in medical education assessment. The resulting model highlights a four-layer structure, illustrating which mega-opportunities serve as foundational drivers and which depend more heavily on upstream developments. This mapping offers a strategic lens/roadmap for educators and researchers to prioritise interventions and understand how advancing one opportunity might trigger progress across others to boost the application of AI in medical education assessment. Figure 2 shows the interdependency model amongst those seven mega-opportunities.

As could be found in Fig. 2, the ISM analysis produced a four-layered hierarchical model that illustrates the significant interdependencies among the seven identified mega-opportunities in AI-driven student assessment within the field of medical education. The model, which should be read from left to right, organises these opportunities based on their strategic positioning, from foundational drivers to those most influenced by the other mega-opportunities.

At the base of the model (Layer 1), two root opportunities are identified: Automated Feedback and Evaluation and Data-Driven Analytics and Curriculum Improvement. These two mega-opportunities are placed within a shared node, indicating a mutual and significant influence on each other. Their foundational role lies in their capacity to generate real-time, data-informed insights that shape and guide the broader assessment ecosystem. The model suggests that these root opportunities exert a strong causal influence on the rest of the system and should be prioritised for early-stage interventions or strategic investment. Specific AI tools - such as learning analytics dashboards that use predictive models to identify at-risk students - offer valuable opportunities for enhancing assessment in medical education.

Moving forward, these foundational opportunities significantly impact the second layer, which includes Simulation-Based Assessment and Longitudinal Assessment and Development. Positioned as intermediate opportunities, these areas rely on feedback mechanisms and data analytics to enhance the authenticity, continuity, and effectiveness of assessment over time. These two, in turn, act as bridges to the third layer.

Layer 3 comprises connector opportunities: Competency-Based and Skills Assessment and Enhancing Focus, Efficiency, and Time Management. These mega-opportunities are influenced by the upstream simulation and longitudinal systems and are pivotal in operationalising assessment insights into competency tracking, student workflow management, and broader academic development. They serve as functional links that translate complex assessment data into usable, individualised actions.

Finally, at the right side of the hierarchy (Layer 4), the model positions Personalised Learning and Adaptive Assessment as an impact opportunity. This placement reflects its high level of dependency on all preceding layers. While it represents one of the most promising and student-centred applications of AI, its realisation is contingent upon the successful integration and functioning of all earlier mega-opportunities in the model. It embodies the cumulative effect of enhanced feedback systems, longitudinal tracking, simulation fidelity, and competency analysis.

Overall, this layered model provides a strategic roadmap for educators, researchers, and policymakers by identifying leverage points and sequencing priorities for the integration of AI in medical education assessment. Additional applications of this roadmap are explored in the next section of the manuscript.

## Discussion

The ISM-based model developed in this study offers more than a static mapping of AI-driven assessment opportunities; it reveals a strategic and systematic hierarchy of leverage points that should inform both future research and practical implementation of AI tools in medical education. The four-layered structure - root, intermediate, connector, and impact opportunities - demonstrates that the transformative potential of AI in assessment lies not in the technology itself, but in how various innovations are sequenced, integrated, and scaffolded across the educational system.

Critically, the emergence of “Automated Feedback and Evaluation” and “Data-Driven Analytics and Curriculum Improvement” as root opportunities challenges the common tendency in medical education to view AI primarily as a tool for content delivery or assessment efficiency. Instead, this model positions these elements as foundational enablers of educational intelligence, supporting adaptive learning ecosystems rather than isolated interventions. However, while these capabilities offer significant promise - particularly in terms of scalability and consistency - they also raise important concerns about reliability, transparency, and trustworthiness. As highlighted in recent policy guidance by UK Parliament [10], the accuracy and fairness of AI-generated insights must be carefully scrutinised, particularly when applied to high-stakes educational settings. This underscores the importance of embedding AI within robust learning analytics infrastructures that not only support educational transformation but also uphold ethical and methodological rigour [99].

Moreover, the cascading influence from root to impact opportunities highlights a profound interdependence between formative and summative assessment strategies. Simulation and longitudinal assessment, positioned as intermediary elements, illustrate how contextualised, experiential, and continuous evaluation can effectively bridge these two assessment approaches. By linking real-time formative feedback with longer-term summative outcomes, they contribute to a more cohesive and learner-centred assessment ecosystem. This insight suggests that meaningful adoption of AI in medical education assessment should be developmental rather than transactional to support learners over time and across diverse learning contexts, rather than concentrating solely on isolated test performance.

Particularly striking is the placement of Personalised Learning and Adaptive Assessment at the apex of the four-layered model as the most dependent, rather than initiating, opportunity. While personalised learning is often championed as the primary promise of AI in education, the ISM model reveals that its effectiveness is contingent upon a robust infrastructure and smart implementation of analytics, feedback, simulation, and competency-tracking. This challenges techno-centric discourses that frame personalisation as a direct output of machine learning models (for example in MOOCs), instead arguing for a systems-level, pedagogically grounded approach [100, 101].

From a theoretical standpoint, the model affirms a sociotechnical and ecological view of innovation in medical education assessment. It underscores the importance of moving from a linear adoption of AI tools to a networked, integrated, coherent, and layered logic of educational change, where technology, pedagogy, and assessment evolve in mutual reinforcement. This echoes the growing call in educational AI scholarship for designing AI within pedagogical ecosystems, rather than inserting it into pre-existing frameworks [102–104].

To illustrate the roadmap’s practical application, consider a medical school’s assessment team aiming to strategically integrate AI. Instead of adopting an isolated AI tool for a single purpose, the team could use our roadmap to prioritise their efforts. The model would guide them to first focus on root opportunities, such as piloting Automated Feedback and Evaluation in low-stakes formative quizzes to build faculty trust and validate the technology’s accuracy. Once these foundational elements are established, the team could then progress to intermediate opportunities, integrating AI into Simulation and Longitudinal Assessment to create more cohesive and continuous evaluation systems. Finally, by building on this robust infrastructure, the team would be equipped to effectively implement the impact opportunities, such as Personalised Learning and Adaptive Assessment, which are revealed to be the most dependent and complex to achieve. This strategic, layered approach moves the institution beyond ad-hoc AI adoption, enabling a systematic and pedagogically grounded transformation of its assessment practices.

Finally, the model helps illuminate a major gap in the literature: while many studies report on discrete applications of AI in medical education assessment, few offer a coherent architecture that reveals how those innovations relate to each other and which offer the greatest strategic leverage. This study responds to that gap by not only categorising existing AI opportunities for medical education assessment but also modelling their cause-effect interdependencies, thus offering a roadmap for research initiatives and funding, curricular innovation, and policy prioritisation.

## Conclusion

This study has taken a critical step toward addressing the fragmented nature of existing research on the role of AI in student assessment within medical education [105, 106] by offering a structured, multi-layered roadmap. Through a comprehensive literature review, categorisation of 22 AI-driven assessment opportunities, and ISM of their cause-effect interdependencies, this research has generated a strategic framework that not only organises current knowledge but also prioritises high-impact innovation areas. The proposed four-layer model - comprising root, intermediate, connector, and impact opportunities - serves as both a diagnostic tool for understanding the current landscape and a developmental guide for shaping future research and implementation strategies [107, 108].

While our ISM model provides a strategic roadmap for adopting AI in medical education assessment, its practical implementation requires careful consideration of institutional context and ethical principles. A phased piloting approach is recommended, beginning with the foundational ‘root’ opportunities like automated feedback in low-stakes formative settings to build stakeholder trust and refine institutional workflows. As institutions progress along the roadmap, particular attention must be paid to the ethical, trust, and equity implications, especially where AI is integrated into high-stakes, summative assessments. This involves establishing clear policies for algorithmic transparency, ensuring datasets used for model training are free from bias, and providing robust mechanisms for human oversight and appeal. Upholding these principles is not just a matter of compliance; it is fundamental to ensuring that AI-driven innovations in medical education are not only effective but also fair, equitable, and trustworthy for all learners. This roadmap, therefore, serves as a guide for both pedagogical innovation and responsible governance, advocating for an implementation strategy that is as thoughtful as it is transformative [109].

Importantly, the roadmap presented here is not intended as a static or prescriptive solution. Rather, it provides a living framework that can evolve with advancements in AI, pedagogical theory, and educational technology. Future studies may refine and expand this model by validating it across different medical education contexts, incorporating diverse stakeholder perspectives (including academics, students and patients), and exploring potential ethical, regulatory, and cultural dimensions [110, 111]. Moreover, institutions and policymakers can adopt this roadmap as a strategic lens to guide investment, curriculum redesign, and faculty development initiatives, ensuring that AI integration into assessment is both impactful and responsible [112]. As such, this study contributes not only to academic scholarship but also to the systemic transformation of assessment practices in medical education.

Despite the valuable contributions of this study, several limitations should be acknowledged. The categorisation and modelling of AI-driven assessment opportunities were based on a purposive sample of expert opinions and existing literature, which, while rich in insight, may not capture the full diversity of global practices or emerging innovations in medical education. Furthermore, the ISM method identifies significant interdependencies but does not quantify their strength or intensity [113], which future studies using complementary methodologies (e.g., Equational Structural Modelling: ESM) could address. Practically, the proposed roadmap offers medical schools and educational technology developers a strategic framework for prioritising AI-driven innovations in assessment, supporting more personalised, scalable, and competency-aligned educational systems. From a research perspective, this study lays the groundwork for longitudinal investigations into the real-world impact of each mega-opportunity on student learning, professional preparedness, and educational equity. Future research should also explore how this roadmap adapts across diverse educational contexts and evolves in response to rapid advancements in AI capabilities. 

## Data Availability

All data generated or analysed during this study are included in this published article. Additional details or clarifications related to the methodology and data are available from the corresponding author upon reasonable request. The data are not publicly deposited in a third-party repository but can be shared upon request, subject to reasonable data sharing and copyright considerations.
